# Supplementary material for: Empirical methods for controlling false positives and estimating confidence in ChIP-Seq peaks
Source: BMC Bioinformatics. 2008 Dec 5;9:523. doi: 10.1186/1471-2105-9-523 (PMC2628906; doi:10.1186/1471-2105-9-523)
Supplement: Additional File 1 — A variety of html documents from the USeq web site detailing the available applications, their best usage, output file type descriptions, command line menus, etc. [file 1471-2105-9-523-S1.zip › USeqUserGuides/JavaDoc.zip!/JavaDoc/meme/MotifHit.html]

MotifHit


|  |  |  |
| --- | --- | --- |
| **Overview** \| **Package** \| **Class** \| **Use** \| **Tree** \| **Deprecated** \| **Index** \| **Help** | |  |
| **PREV CLASS**   **NEXT CLASS** | **FRAMES**    **NO FRAMES**     **All Classes** |  |
| SUMMARY: NESTED \| FIELD \| CONSTR \| METHOD | DETAIL: FIELD \| CONSTR \| METHOD |  |


---


## meme Class MotifHit

```
java.lang.Object
  meme.MotifHit
```

**All Implemented Interfaces:**: java.lang.Comparable

---

``` public class MotifHit extends java.lang.Object implements java.lang.Comparable ```

Holds data related to a particular match of a matrix to a sequence. Interbase coordinates.

---

**Constructor Summary**

`MotifHit(int st,
int sp,
double sc,
java.lang.String s,
int o)`


| **Method Summary** | |
| --- | --- |
| `int` | `compareTo(java.lang.Object otherObject)` |
| `int` | `getOrientation()` |
| `double` | `getScore()` |
| `java.lang.String` | `getSeq()` |
| `int` | `getStart()` |
| `int` | `getStop()` |
| `void` | `setScore(double score)` |
| `void` | `setSeq(java.lang.String seq)` |
| `void` | `setStart(int start)` |
| `void` | `setStop(int stop)` |
| `java.lang.String` | `toString()` |

**Methods inherited from class java.lang.Object**

`equals, getClass, hashCode, notify, notifyAll, wait, wait, wait`

**Constructor Detail**

### MotifHit

```
public MotifHit(int st,
                int sp,
                double sc,
                java.lang.String s,
                int o)
```


**Method Detail**

### compareTo

```
public int compareTo(java.lang.Object otherObject)
```

:   **Specified by:**: `compareTo` in interface `java.lang.Comparable`

---


### toString

```
public java.lang.String toString()
```

:   **Overrides:**: `toString` in class `java.lang.Object`

---


### getScore

```
public double getScore()
```

---


### setScore

```
public void setScore(double score)
```

---


### getSeq

```
public java.lang.String getSeq()
```

---


### setSeq

```
public void setSeq(java.lang.String seq)
```

---


### getStart

```
public int getStart()
```

---


### setStart

```
public void setStart(int start)
```

---


### getStop

```
public int getStop()
```

---


### setStop

```
public void setStop(int stop)
```

---


### getOrientation

```
public int getOrientation()
```


---


|  |  |  |
| --- | --- | --- |
| **Overview** \| **Package** \| **Class** \| **Use** \| **Tree** \| **Deprecated** \| **Index** \| **Help** | |  |
| **PREV CLASS**   **NEXT CLASS** | **FRAMES**    **NO FRAMES**     **All Classes** |  |
| SUMMARY: NESTED \| FIELD \| CONSTR \| METHOD | DETAIL: FIELD \| CONSTR \| METHOD |  |


---
